# Assessment of the effect of perineural dexmedetomidine on oxidative stress during peritoneal dialysis catheter insertion: a randomized, controlled trial

**DOI:** 10.1007/s11255-022-03268-4

**Published:** 2022-06-30

**Authors:** Xiaoli Zhang, Guangsen Liu, Cong Sun, Yi Zhong, Ling Wang, Zhihua Huang, Guoping Wang, Reai Shan

**Affiliations:** 1 First Clinical Medical College, Gannan Medical University, Ganzhou, China; 2 Key Laboratory of Prevention and Treatment of Cardiovascular and Cerebrovascular Diseases of Ministry of Education, Gannan Medical University, Ganzhou, China; 3 Department of Nephrology, First Affiliated Hospital of Gannan Medical University, Ganzhou, China; 4 Department of Anesthesiology, First Affiliated Hospital of Gannan Medical University, Pain Institute, Gannan Medical University, Ganzhou, China; 5 Pain Institute, Gannan Medical University, Ganzhou, China

**Keywords:** Dexmedetomidine, End-stage renal disease, Oxidative stress, Peritoneal dialysis, Ropivacaine

## Abstract

**Purpose:**

This study aimed to evaluate the effect of the addition of dexmedetomidine to ropivacaine on oxidative stress during transversus abdominis plane (TAP) and rectus sheath (RS) blockades for patients with end-stage renal disease (ESRD) undergoing peritoneal dialysis (PD) catheter insertion.

**Methods:**

Sixty patients with ESRD undergoing PD catheter insertion to receive left ultrasound-guided TAP and RS blockades were randomly divided into two groups: the dexmedetomidine plus ropivacaine group (25 mL of 0.3% ropivacaine + 1 μg/kg dexmedetomidine) and the ropivacaine group (25 mL of 0.3% ropivacaine). Primary outcomes were oxidative stress marker levels during the procedure.

**Results:**

A total of 60 patients (30 patients in each group) were evaluated. Compared with the ropivacaine group, the dexmedetomidine plus ropivacaine group had significantly lower serum malondialdehyde levels (*P* < 0.05) and increased glutathione peroxidase (*P* < 0.01) and superoxide dismutase levels at 24 h after the procedure (*P* < 0.01).

**Conclusion:**

The addition of 1 μg/kg of dexmedetomidine to ropivacaine for ultrasound-guided TAP and RS blockades could inhibit oxidative stress in patients with ESRD undergoing PD catheter insertion.

*Trial registration* This study was registered at www.chictr.org.cn on June 7, 2021 (ChiCTR2100047050).

## Introduction

Peritoneal dialysis (PD) is an effective alternative treatment involving PD catheter placement in patients with end-stage renal disease (ESRD) using either open surgical, laparoscopic, or percutaneous approaches [1]. At present, this surgical technique remains the most widely used method in clinical practice in China; it is accurate and reliable, involves few complications, and is suitable for the majority of patients who intend to undergo PD. It has been reported in the literature that ultrasound-guided transversus abdominis plane (TAP) and rectus sheath (RS) blockades can be used for open surgical PD catheter insertion [2]. However, because of the lack of sedation and visceral analgesia, propofol and sufentanil consumption were increased during surgery; without satisfactory sedation and visceral analgesia, the surgical process may be affected [3].

Surgery is an acute event that classically results in a “stress response” driven by endocrine changes that lead to metabolic sequelae that have a close association with oxidative stress (OS) [4]. During the past 2 decades, OS has emerged as a novel risk factor for various adverse events. Furthermore, OS is considered an important novel and nontraditional risk factor contributing to increased mortality and cardiovascular morbidity for ESRD population [5].

Dexmedetomidine, a highly selective a_2_ adrenergic agonist with sedative, analgesic, and antioxidant effects, has been used for PD catheter insertion to safely improve operative analgesia and provide potential renal-protective effects [6–8]. Recently, dexmedetomidine has been safely and effectively used with regional blockades to potentiate the analgesic properties of ropivacaine, reduce sufentanil consumption, and provide protective effects without any major side effects [9]. The anti-OS effects of dexmedetomidine have been highlighted recently. ESRD is characterized by its pro-oxidant status caused by the imbalance between antioxidative and oxidative mechanisms [10]. Preclinical murine acute stress models demonstrated that dexmedetomidine significantly improved renal dysfunction, ameliorated kidney injury, and reduced OS; furthermore, studies have shown that intravenous dexmedetomidine effectively reduced stress hormone release and attenuated the surgical stress response without untoward hemodynamic adverse events [11]. However, the effects of dexmedetomidine, which is used in addition to local anesthetics during surgery, on the inhibition of the OS response have not been evaluated.

Therefore, during this prospective, randomized, double-blind study, we investigated whether the use of ropivacaine combined with dexmedetomidine for left TAP and RS blockades could suppress the stress response in patients undergoing PD catheter placement. The primary outcome of the study was the change in stress marker levels during the procedure. The secondary outcomes included hemodynamic changes, pain scores, sedation scores, sensory blockade duration, and adverse events.

## Methods

### Study design and participants

This prospective, randomized trial was approved by the Medical Ethics Committee of First Affiliated Hospital of Gannan Medical University (LLSC-2020101403). This study was registered at www.chictr.org.cn on June 7, 2021 (ChiCTR2100047050). Written informed consent was obtained from all patients who participated in this study. The study was conducted and reported in accordance with the Consolidating Standards of Reporting Trials (CONSORT) 2010 statement. Sample size estimates were based on previous experience. The mean serum concentration of superoxide dismutase (SOD) in rats was 30.87 U/mg (standard deviation, 6.47). Assuming a two-sided test with an error of 0.05, a b error of 0.2, and a dropout rate of 10%, 17 patients were required in each group to detect a difference of ≥ 20% in the mean values of the two groups [12].

PD catheter placement using ultrasound-guided left TAP and RS blockades as the primary anesthetic modality was performed for 60 patients with ESRD from July 2021 to December 2021. Patients with American Society of Anesthesiologists physical status 3 or 4 were included in the study. Exclusion criteria included refusal to participate, coagulation disorders, allergy to local anesthetic, localized infection at the injection site, contraindications to receiving dexmedetomidine, and morbid cardiovascular impairments, including preexisting heart blockade or compromised left ventricular function (defined as ejection fraction < 45%). Patients who were unable to communicate were also excluded.

### Randomization and minimization of bias

Patients were randomly assigned to receive either ropivacaine (R group) or 1 µg/kg dexmedetomidine plus ropivacaine (DR group) based on a computer-generated table of random numbers. All patients were blinded. Group allocation was concealed using a sequentially numbered, sealed, opaque envelope that was opened by a separate investigator who prepared the anesthetic solution before surgery. The procedure was performed by a single experienced surgeon (blinded to the study protocol) using the same technique for every patient.

### Anesthetic procedures

Intravenous access was secured before arrival to the operating room. In the operating room, standard monitoring was applied. The DR group received 10 mL (75 mg) ropivacaine 7.5 mg/mL plus 1 µg/kg dexmedetomidine and 0.9% saline (total of 25 mL). The R group received 10 mL (75 mg) ropivacaine 7.5 mg/mL and 0.9% saline (total of 25 mL).

The anesthetic procedures were performed by a single experienced anesthesiologist (blinded to the study protocol) using the same technique for every patient. The TAP blockade was performed using an in-plane technique guided by real-time ultrasound sonography with a 9-cm 22-G needle (KDL Medical Company, Zhejiang, China). After the tip of the needle was advanced to the correct neurovascular plane between the internal oblique and transversus abdominis and negative aspiration was confirmed, the 15-mL solution was administered with direct ultrasonographic guidance. The RS blockade was performed at the junction of the lower edge of the left rib arch and lateral margin of the rectus abdominis muscle. The needle was inserted in the plane to the transducer in a medial to lateral direction, with the endpoint being the fascial plane between the rectus abdominis muscle and posterior RS. After the tip of the needle was advanced to the correct plane and negative was aspiration confirmed, the 10-mL solution was administered using direct ultrasonographic guidance. The effects of the TAP and RS blockades were evaluated using a cold sensation test and by testing whether pain was felt with a pinprick. At least 10 min were allowed to elapse after completing the blockade before the skin incision was initiated. If the patients felt significant pain at the time of the incision, then 5 to 10 mL of lidocaine was injected as rescue analgesic.

### Outcome assessments

Data were recorded at the following time points: *T*_0_, baseline; *T*_1_, after the TAP and RS blockades; *T*_2_, at the beginning of surgery; *T*_3_, when a small hole was created in the parietal peritoneum; *T*_4_, insertion of the PD catheter; *T*_5_, tunnel formation; *T*_6_, end of surgery; and *T*_7_, 24 h after surgery.

Venous blood samples were obtained at *T*_0_, *T*_6_, and *T*_7_ to detect serum levels of malondialdehyde (MDA), glutathione (GSH) peroxidase (GSH-Px), and SOD using colorimetric methods. The mean arterial pressure (MAP) and heart rate (HR) were also recorded, and the rate-pressure product (RPP) (RPP = systolic blood pressure × HR) was calculated. Patients were asked to assess their pain level during the procedure using a visual analog scale (VAS) ranging from 0 (no pain) to 10 (worst possible pain) from *T*_2_ to *T*_6_. Patient sedation levels were checked during the surgery based on the observer’s assessment of alertness/sedation (OAA/S) scale (5, responds readily to name spoken in a normal tone; 4, lethargic response to name spoken in a normal tone; 3, responds only after name is spoken loudly or repeatedly; 2, responds after mild prodding or shaking; 1, does not respond to mild prodding or shaking). The duration of the sensory blockade was defined as the time from completing both blockades to the time when the cold sensation in response to alcohol returned.

Intraoperative hypotension (defined as > 30% decrease in systolic pressure from baseline) was treated with intravenous ephedrine (5–10 mg). Bradycardia (defined as heart rate < 45 beats min^–1^) was treated with intravenous atropine (0.5 mg).

### Statistical analysis

The statistical analysis was performed using GraphPad Prism 8 (GraphPad Software, San Diego, CA, USA). Data are expressed as the mean ± standard deviation or the median (interquartile range). Data were analyzed using a repeated-measures analysis of variance. Intergroup differences at the same time point were analyzed using a two-sample t test. *P* < 0.05 was considered statistically significant.

## Results

**Fig. 1:**
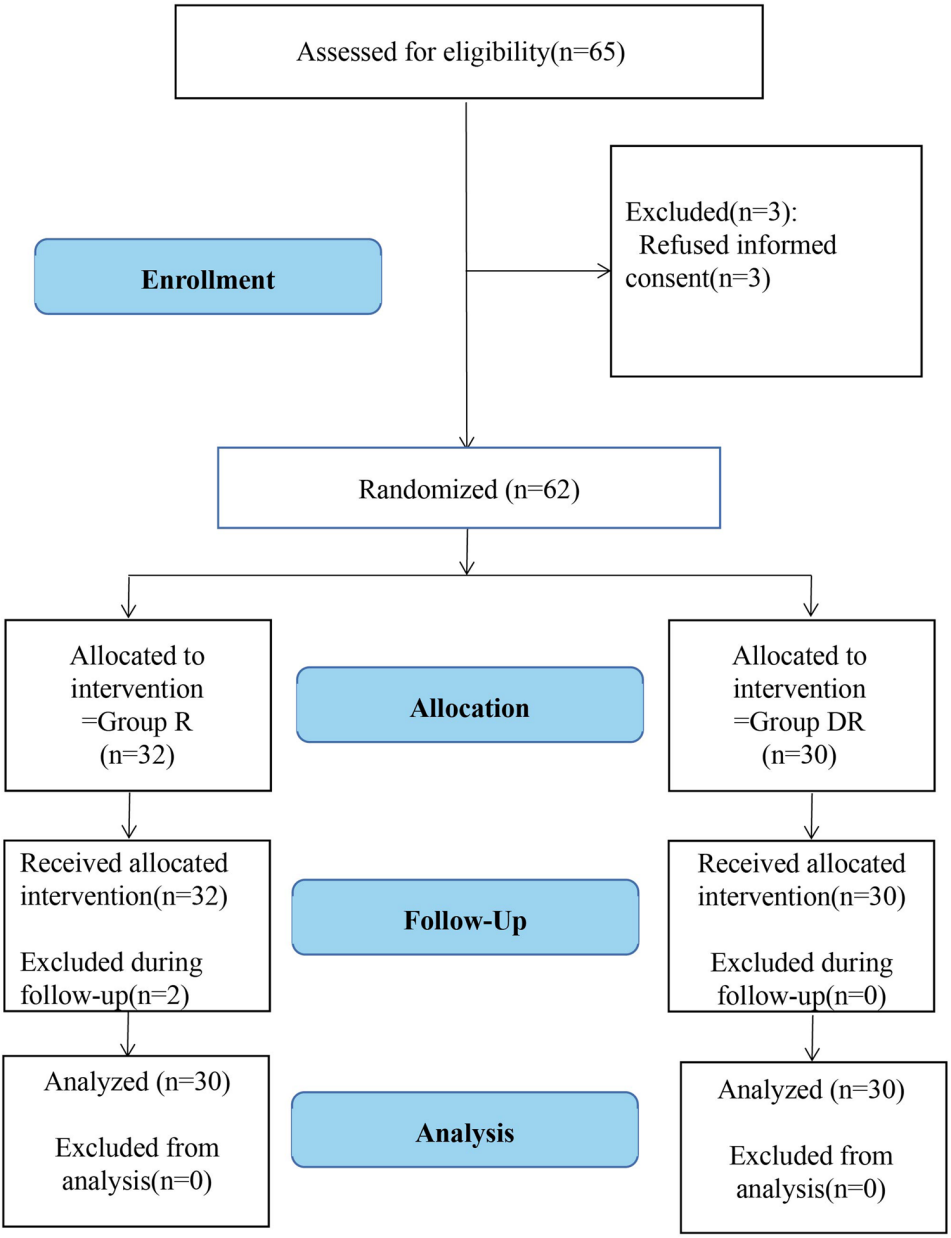
CONSORT flow diagram for the study

Initially, a total of 65 patients were enrolled; however, three patients refused to provide informed consent. Two patients in the R group were excluded because of their assessments regarding the primary outcome were incomplete. Therefore, 60 subjects completed the study and were analyzed (Fig. 1). Baseline characteristics of the included patients were comparable between the groups (Table 1).

**Table 1:** Demographic data and surgical data

	Group R (*n* = 30)	Group DR (*n* = 30)	*P* value
Gender, M/F	13/17	15/15	0.6048
Age, years	43.267 ± 13.04	46.933 ± 11.55	0.2538
Weight, kg	57.29 ± 9.72	56.34 ± 11.81	0.7349
ASA, (III/IV)	21/9	24/6	0.3711
Operation time, min	73.43 ± 37.11	63.57 ± 24.55	0.2295

No statistically significant difference was observed between groups

Group R: ropivacaine alone; Group DR: 1 µg/kg dexmedetomidine plus ropivacaine

### Primary outcomes

**Fig. 2:**
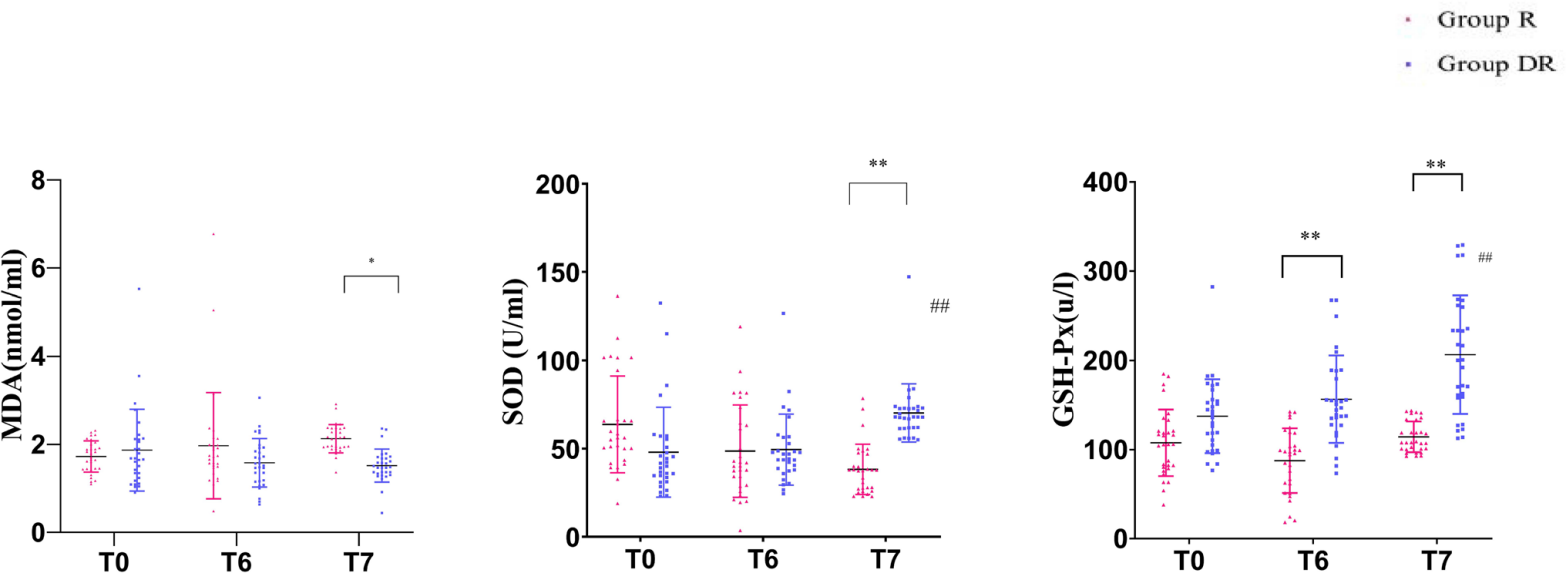
Serum concentration of MDA, SOD, and GSH-Px. Compared with Group R, *P < 0.05;**P < 0.01; versus *T*_0_ of group DR, ^##^*P* < 0.01. *T*_0_, baseline; *T*_6_, end of surgery; *T*_7_, 24 h after surgery. Group R: ropivacaine alone; Group DR: 1 µg/kg dexmedetomidine plus ropivacaine

There were no significant differences between groups regarding SOD, MDA, and GSH-Px at baseline (*P* > 0.9999). As shown in Fig. 2, serum MDA levels were not statistically different at *T*_0_ or *T*_6_ in both groups. However, a significant increase in MDA was observed in the R group, but not in the DR group, at *T*_7_ (*P* < 0.05), indicating that dexmedetomidine prevented the increase in serum MDA levels. Similarly, serum SOD levels were not statistically different at *T*_0_ and *T*_6_ in both groups; however, serum SOD levels of the DR group were significantly higher than those of the R group at *T*_7_ (*P* < 0.01). In addition, the serum SOD level at *T*_7_ of the DR group was higher than that at *T*_0_ (*P* < 0.01). A significant increase in GSH-Px was observed in the DR group at *T*_6_ and *T*_7_ (*P* < 0.01).

### Secondary outcomes

To assess the effects of analgesia, the VAS scale was analyzed from *T*_2_ to *T*_6_. Compared with those of the R group, the VAS scores of the DR group were significantly lower at *T*_4_ (*P* < 0.001); there was no significant difference between groups at the other time points (*P* > 0.05) (Table 2).

**Table 2:** Comparison of visual analog scale

	Group R (*n* = 30)	Group DR (*n* = 30)	*P* value
*T*_2_	1.233 ± 1.675	1.467 ± 1.717	0.9838
*T*_3_	0.067 ± 0.365	0.033 ± 0.183	> 0.999
*T*_4_	7.367 ± 1.884	4.600 ± 3.001	< 0.01**
*T*_5_	1.333 ± 1.295	1.067 ± 1.856	0.9709
*T*_6_	0.100 ± 0.403	0.167 ± 0.648	> 0.999

Values are presented as mean ± standard deviation; R: ropivacaine alone; DR: 1 µg/kg dexmedetomidine plus ropivacaine; *T*_2_, at the beginning of surgery; *T*_3_, when a small hole was created in the parietal peritoneum; *T*_4_, insertion of the PD catheter; *T*_5_, tunnel formation; *T*_6_, end of surgery; **P < 0.01 versus Group R

There was no significant difference between groups in the OAA/S scales at baseline (*P* > 0.9999). Compared with the R group, the OAA/S scales were significantly lower from *T*_1_ to *T*_6_ (*P* < 0.001) (Table 3).

**Table 3:** Comparison of observer’s assessment of alertness/sedation

	Group R (*n* = 30)	Group DR (*n* = 30)	*P* value
*T*_0_	5.000 ± 0.000	5.000 ± 0.000	> 0.999
*T*_1_	5.000 ± 0.000	3.467 ± 1.042	< 0.01**
*T*_2_	5.000 ± 0.000	2.300 ± 1.149	< 0.01**
*T*_3_	5.000 ± 0.000	1.367 ± 0.765	< 0.01**
*T*_4_	5.000 ± 0.000	1.933 ± 1.015	< 0.01**
*T*_5_	5.000 ± 0.000	1.833 ± 0.986	< 0.01**
*T*_6_	5.000 ± 0.000	3.467 ± 1.358	< 0.01**

Values are presented as mean ± standard deviation; R: ropivacaine alone; DR: 1 µg/kg dexmedetomidine plus ropivacaine; *T*_0_, baseline; *T*_1_, after the TAP and RS blockades; *T*_2_, at the beginning of surgery; *T*_3_, when a small hole was created in the parietal peritoneum; *T*_4_, insertion of the PD catheter; *T*_5_, tunnel formation; *T*_6_, end of surgery; **P < 0.01 versus Group R

There were no significant changes in MAP and HR over time. There were no significant differences between groups at any time point (*P* > 0.05). There were no significant differences in the RPP between groups from *T*_0_ to *T*_2_ (*P* > 0.05). The RPP was significantly lower in the DR group from *T*_3_ to *T*_6_ (*P* < 0.05) (Table 4).

**Table 4:** Changes in MAP, HR and RPP

Time point	MAP (mmhg)			HR (bpm)			RPP (mmhg/min)		
	Group R	Group DR	*P* value	Group R	Group DR	*P* value	Group R	Group DR	*P* value
*T*_0_	111.00 ± 15.23	109.07 ± 16.92	0.9987	77.00 ± 13.70	80.37 ± 12.13	0.9552	12,618.73 ± 3246.80	12,607.23 ± 2687.91	> 0.9999
*T*_1_	112.37 ± 14.21	109.87 ± 15.28	0.9936	75.67 ± 14.86	76.13 ± 13.21	> 0.9999	12,326.83 ± 2969.42	11,605.60 ± 2691.91	0.9294
*T*_2_	112.73 ± 13.69	107.10 ± 13.08	0.6571	77.77 ± 15.63	74.07 ± 12.77	0.9276	12,450.27 ± 2993.83	10,972.90 ± 2519.93	0.2484
*T*_3_	108.53 ± 13.65	105.30 ± 13.08	0.9716	76.13 ± 14.65	70.53 ± 11.65	0.6130	11,833.63 ± 2699.55	9952.90 ± 2255.18^#^	0.0618
*T*_4_	108.07 ± 14.61	103.23 ± 15.29	0.8033	76.67 ± 16.06	69.87 ± 12.46	0.3703	11,816.63 ± 3185.53	9271.87 ± 1908.67^#^	0.0024*
*T*_5_	107.53 ± 16.65	102.77 ± 15.96	0.8141	77.37 ± 17.96	69.60 ± 12.56	0.2177	11,952.10 ± 3537.09	9607.40 ± 1985.75^#^	0.0082*
*T*_6_	109.80 ± 15.66	102.43 ± 12.44	0.3266	75.20 ± 16.84	68.53 ± 12.20	0.3951	11,865.04 ± 3411.53	9526.40 ± 2187.29^#^	0.0102*

Values are presented as mean ± standard deviation; R: ropivacaine alone; DR: 1 µg/kg dexmedetomidine plus ropivacaine; *T*_0_, baseline; *T*_1_, after the TAP and RS blockades; *T*_2_, at the beginning of surgery; *T*_3_, when a small hole was created in the parietal peritoneum; *T*_4_, insertion of the PD catheter; *T*_5_, tunnel formation; *T*_6_, end of surgery; no statistically significant difference was observed in MAP and HR between groups. *P < 0.05 versus Group R; ^#^*P* < 0.05 versus *T*_0_ in group DR

The durations of the sensory blockades in the DR group and R group were 458.87 ± 259.00 min and 274.00 ± 156.84 min, respectively, indicating that dexmedetomidine prolongs the analgesia duration (*P* = 0.0082).

No adverse reactions, such as sinus bradycardia, hypotension, respiratory depression, and local anesthetic intoxication, were observed in either group. No nausea or vomiting occurred in either group.

## Discussion

Our study demonstrated that ultrasound-guided TAP and RS blockades with ropivacaine and 1 µg/kg dexmedetomidine provide satisfactory analgesia and sedation effects and prolong the analgesia duration to relieve OS in patients with ESRD during PD catheter insertion without side effects.

Because of the associated comorbidities, providing anesthesia to patients requiring PD catheter insertion is a real challenge to anesthesiologists; furthermore, there are many restrictions to the choice of general anesthesia and spinal anesthesia for patients with ESRD. Regional anesthesia became popular because of its satisfactory anesthetic effect and negligible systemic effects, especially for patients with significant comorbidities. Previous studies have shown that TAP and RS blockades can provide effective analgesia during PD catheter placement, and they have been shown to be an ideal anesthetic option for patients with ESRD [2, 3, 13]. However, ultrasound-guided TAP and RS blockades with ropivacaine individually cannot relieve the pain of PD catheter insertion and anxiety caused by the procedure that result in perioperative OS. Intraoperative anesthesia and surgical stimulation can upset the balance of oxygen free radicals and oxidative defense, leading to OS damage to the organism [14].

OS occurs during the early stages of chronic kidney disease and increases in parallel with the progression of renal impairment; it is much higher with ESRD [15]. Previous studies suggested that OS might have a role in the pathogenesis and prognosis of PD peritonitis, which promotes the overproduction of pro‐oxidant molecules and is accompanied by dramatic structural and functional damage to peritoneal membrane cells [16]. In addition, preservation of the residual renal function has been closely associated with survival, and enhanced OS strongly predicts deterioration in the residual renal function [17]. As a result, reducing perioperative OS is essential for patients with ESRD. SOD is an antioxidant enzyme present in the body that has an eliminating effect on harmful substances produced during the metabolism of living organisms, and it is the most important free radical scavenger in the body because it catalyzes the disproportionation of superoxide anions [18]. MDA is a low-molecular-weight aldehyde that originates from the breakage of lipid peroxide radical, and it is an indicator of OS and lipid peroxidation; when lipid peroxidation occurs in cells, MDA will be generated. Therefore, it can be used as an important indicator of OS in the body, and the amount generated is proportional to the degree of peroxidation, which can reflect the degree of cell damage. GSH-Px is an important enzyme that is widely present in the body and catalyzes the breakdown of hydrogen peroxide; the direct antioxidant action of GSH is guided by the activity of GSH-Px [19]. It specifically catalyzes the reduction of hydrogen peroxide with reduced GSH, which can have a role in protecting the structural and functional integrity of cell membranes, and it is an important factor involved in the repair of damaged DNA during DNA synthesis [20]. The impact of preoperative risk factors and the propagation of intraoperative OS are often confirmed in the postoperative period and may manifest as unnecessary complications and long-term harm. A small but growing number of clinical studies have shown a positive correlation between high OS in the perioperative period and postoperative complications in patients undergoing major surgery, including liver, lung resections, and cardiac surgery. The use of OS in predicting long-term outcomes may also be feasible [21–24].

The use of dexmedetomidine is becoming more widespread because of its anti-OS effects. Previous studies have shown that the addition of dexmedetomidine to perineural local anesthetics alleviates OS in rats; however, relevant clinical studies are rare [25–27]. During this study, MDA in the DR group was significantly lower than that in the R group at *T*_7_, and GSH-Px and SOD were significantly higher than those in the R group at *T*_7_. The results of the present study suggest that dexmedetomidine, as a local anesthetic adjuvant, can have an anti-OS effect, which is consistent with the results of animal experiments.

The PD catheter is supposed to be placed in the rectal crypt. The insertion of the catheter can lead to direct contact of the catheter tip with the floor of the pelvic cavity, causing the patient to feel pain or the requirement to defecate. The significantly lower VAS score at *T*_4_ in the DR group demonstrates that the addition of dexmedetomidine could provide satisfactory abdominal viscera analgesia. The analgesic effects of dexmedetomidine are thought to be caused by the stimulation of α_2_ receptors of the nociceptive neurons in the dorsal horn of the spinal cord and the consequent reduction in the release of substance P [28]. In addition, the surgeon can obtain feedback from the patient to confirm the location of the PD catheter under regional anesthesia. Dexmedetomidine exerts its hypnotic action through selective activation of alpha-2 adrenergic receptors in the locus coeruleus and mediates the sedative effects, thereby allowing for better arousable sedation. The OAA/S score significantly decreased from *T*_1_ to *T*_6_ in the DR group, suggesting that dexmedetomidine as an adjunct can effectively achieve sedation, thus relieving traumatic stimulation. Akeju et al. assumed that the altered arousal states induced with the administration of dexmedetomidine neurophysiologically approximate natural sleep; they termed this “biomimetic sleep [29]”. The pharmacological effect that produces biomimetic sleep allows the surgeon to obtain feedback from the patient during the procedure, thereby achieving a balance of analgesia and sedation.

Kidney failure is often accompanied by hypertension and arrhythmias; therefore, maintaining hemodynamic stability is of great importance. It is well known that bradycardia and hypotension are dose-dependent. The best dose in combination with 0.3% ropivacaine for TAP and RS blockades to produce strong inhibition of OS without increasing the incidence of bradycardia and hypotension in patients with ESRD is undetermined [30, 31]. There is strong evidence that dexmedetomidine in doses of 50 to 60 μg prolongs and improves the quality of brachial plexus blockades with minimal to no side effects; however, evidence of the benefits of perineural dexmedetomidine for other peripheral nerve blockades is sparse [32]. During this study, 1 µg/kg of dexmedetomidine was used. All patients in the DR group were sedated adequately throughout the surgery, without bradycardia or hypotension. Although HR and MAP were not statistically different between groups, the RPP was statistically different. Previous studies reported that the RPP was one of the major determinants of myocardial oxygen consumption, and that an RPP more than 20,000 mmHg/min could cause angina pectoris [33]. No patient had an RPP more than 20,000 mmHg/min during our study. The ability of dexmedetomidine to reduce the RPP could decrease the myocardial oxygen requirement and may be advantageous for patients with ESRD.

Dexmedetomidine has been demonstrated to prolong analgesia when used in peripheral nerve blockades, and our study confirmed this [33]. The addition of dexmedetomidine reduced OS, optimized the neural microenvironment, and inhibited norepinephrine release, thus enhancing the neural blockade effectiveness of ropivacaine and resulting in the blockade of nociceptive pain signal transmission. Dexmedetomidine also causes inhibition of cationic neural hyperpolarization, which increases the duration of neural blockades [34].

This study had several limitations. First, we did not determine the optimal dose of dexmedetomidine; therefore, more studies are needed to determine the optimal dexmedetomidine dose. Second, the total amount of dexmedetomidine was calculated based on actual body weight instead of ideal body weight. Moreover, we did not consider the amount of lidocaine as rescue analgesic. Finally, we did not conduct long-term follow-up of patients in this study to elucidate the impact of perioperative oxidative stress on long-term prognosis.

In conclusion, the addition of 1 μg/kg dexmedetomidine to ropivacaine for ultrasound-guided TAP blockade combined with RS blockade for patients with ESRD undergoing peritoneal dialysis catheter insertion effectively suppressed perioperative OS, had little effect on MAP and HR, and reduced the RPP. It provided satisfactory analgesia and sedation effects and prolonged the analgesia duration. Future studies are needed to evaluate the ideal dose of dexmedetomidine that should be added for patients with ESRD undergoing PD catheter insertion.
